# SDF-1 Chemokine Signalling Modulates the Apoptotic Responses to Iron Deprivation of Clathrin-Depleted DT40 Cells

**DOI:** 10.1371/journal.pone.0106278

**Published:** 2014-08-27

**Authors:** Alena Pance, Frank R. Morrissey-Wettey, Helen Craig, Alison Downing, Richard Talbot, Antony P. Jackson

**Affiliations:** 1 Department of Biochemistry, Hopkins Building, University of Cambridge, Cambridge, United Kingdom; 2 Roslin Institute, Roslin, Midlothian, Scotland, United Kingdom; 3 The Wellcome Trust Sanger Institute, Hinxton, Cambridge, United Kingdom; Cedars-Sinai Medical Center, United States of America

## Abstract

We have previously deleted both endogenous copies of the clathrin heavy-chain gene in the chicken pre B-cell-line DT40 and replaced them with clathrin under the control of a tetracycline-regulatable promoter (Tet-Off). The originally derived cell-line DKO-S underwent apoptosis when clathrin expression was repressed. We have also described a cell-line DKO-R derived from DKO-S cells that was less sensitive to clathrin-depletion. Here we show that the restriction of transferrin uptake, resulting in iron deprivation, is responsible for the lethal consequence of clathrin-depletion. We further show that the DKO-R cells have up-regulated an anti-apoptotic survival pathway based on the chemokine SDF-1 and its receptor CXCR4. Our work clarifies several puzzling features of clathrin-depleted DT40 cells and reveals an example of how SDF-1/CXCR4 signalling can abrogate pro-apoptotic pathways and increase cell survival. We propose that the phenomenon described here has implications for the therapeutic approach to a variety of cancers.

## Introduction

Clathrin plays a fundamental role in membrane trafficking pathways in eukaryotic cells. It is responsible for receptor-mediated endocytosis of selected molecules from the plasma membrane and the transport of some lysosomal enzymes from the *trans*-Golgi network to the lysosome [1]. The chicken pre-B cell-line DT40 was originally isolated as an avain-leukosis virus-derived chicken B-lymphocyte cell-line [2]. It exhibits an extraordinarily high rate of homologous recombination, and this property greatly facilitates gene targeting [3]. The DT40 cell-line has thus become established as an important tool for the study of a wide range of cell-biological phenomena [4]. To understand the function of clathrin in more detail, we inactivated both endogenous copies of the clathrin gene in DT40 and replaced them with clathrin under the control of the Tet-Off inducible promoter. In the presence of doxycycline, clathrin expression was strongly inhibited and this allowed the functional consequences of clathrin-depletion to be investigated in a vertebrate cell context. The originally generated cell-line conditionally deficient in clathrin expression was designated DKO-S (*d*ouble *k*nock*o*ut cells, *s*ensitive to clathrin-depletion). In this cell-line, we found that clathrin-depletion induced apoptosis. We also described a variant derived from DKO-S that did not die in the absence of clathrin. We designated this cell-line DKO-R (*d*ouble *k*nock*o*ut cells *r*esistant to clathrin-depletion) [5]. We have used both of these DKO cell-lines to study clathrin function [5], [6], [7], [8], [9].

The apoptotic response of clathrin-depleted DT40 cells is particularly curious. Clathrin expression has been dramatically reduced in other vertebrate cell-lines such as HeLa cells without the activation of apoptosis, or indeed without inducing any form of cell death [10]. Why then does clathrin-depletion in DT40 cells differ? The distinct phenotype of the DKO-R variant raises further questions. What adaptations has this variant acquired to overcome the apoptotic response to clathrin-depletion shown by its DKO-S parent? Here we show that iron deficiency is a major apoptotic signal stemming from clathrin depletion. We furthershow that the resistant cell-line DKO-R has increased survival under these conditions due to endogenous expression of the chemokine SDF-1 and the autocrine stimulation of a survival pathway based on its receptor CXCR-4. We show that this surprisingly small change can explain all the known phenotypic differences between DKO-S and DKO-R cells.

## Materials and Methods

### Cell culture and materials

Except where stated, all reagents were purchased from Sigma, Poole, UK. All cell-lines were maintained in RPMI 1640 media, 0.2% (wt/vol) Na_2_HCO_3_, 1% (wt/vol) L-glutamine with 10% (vol/vol) heat-treated foetal calf serum (FCS) and between 0 and 2% (vol/vol) heat-treated chicken serum as indicated in the text. Serum and media were purchased from Gibco, Paisley, UK. Cells were maintained at 37°C and with 5% CO_2_. Some batches of bovine serum contain the protease dipeptidylpeptidase IV (CD26) that destroys SDF-1 activity [11]. To avoid this complication for experiments using SDF-1, the media was supplemented with the selective CD26 inhibitor diprotin A (0.1 mM) [12]. Cell numbers were counted with a haemocytometer after Trypan Blue staining. Fully iron-saturated chicken transferrin (conalbumin from Sigma) was produced by incubating 10 mgs of protein in 1 ml of 0.1 mg/ml ferric ammonium citrate in phosphate buffered saline (PBS) pH 7.2 for 3 hours at room temperature, then dialysing the protein overnight at 4°C against PBS [13]. Iron-saturated chicken transferrin was stored in 0.1 ml aliquots at −20°C until use. Apotransferrin was produced by dialysing chicken transferrin against two overnight changes of 0.1 M sodium citrate, 0.1 M sodium acetate buffer pH 4.5 at 4°C, followed by an overnight dialysis at 4°C against PBS [14]. Aliquots (0.1 ml) were stored at −20°C until used.

### Electrophoresis and western blotting

Cells were lysed in PBS with 0.1% Triton X-100 (vol/vol) and 1× protease inhibitor cocktail (Boehringer Ingelheim, Bracknell, UK). Total protein was determined using the BCA protein assay kit (Pierce, Cramlington, UK) with bovine serum albumin as standard. Protein (about 50–80 µg cell extract; about 1–2 µg partially purified chicken transferrin) was separated by sodium dodecyl sulphate (SDS) polyacrylamide gel electrophoresis in gels of 8% acrylamide [15]. For western blots, proteins in the gel were transferred to Hybond C membrane (Amersham Biosciences, Chalfont St.Giles, UK). Blots were blocked by incubation for 1 hour in PBS, 0.1% Triton X-100 and 5% powdered milk, then incubated overnight at 4°C with primary antibody (typically 0.1–1 µg/ml) diluted into PBS, 0.1% (vol/vol) Triton X100 and 5% (vol/vol) FCS. Specific signal was detected using horseradish peroxidase-labelled second antibodies and enhanced chemiluminescence (Pierce). To confirm protein loading, nitrocellulose was stained with 0.1% amido black in 10% acetic acid, 45% methanol and destained with 10% acetic acid, 45% methanol. Clathrin was detected using mouse monoclonal antibody TD1 [16]. Chicken transferrin was detected using mouse monoclonal antibodies [17]. Transferrin receptor was detected using mouse monoclonal antibody H68.4 (Invitrogen).

### Apoptosis assays

Cells with apoptotic bodies were counted on a fluorescent microscope following staining with 1 µg/ml Hoechst 33342 [5]. Caspase 3 activity was determined as described [18]. Cells were centrifuged at 12,000 rpm for 8 minutes, washed twice in PBS and lysed in 20 mM HEPES pH 7.4, 10 mM KCl, 250 mM sucrose, 1.5 mm MgCl_2_, 0.5 mM EDTA, 0.5 mM EGTA and protease inhibitor cocktail (Roche, Welwyn Garden City, UK). Samples were incubated with 14 µM acetyl-DEVD-AMC substrate for 40 minutes and fluorescence at 450 nm determined. The rate of reaction was linear over this time period. For inhibitor studies, a 1 mM stock of inhibitor (AMD3100, CCX771 or CCX704) in DMSO was diluted into at final concentration for the assays of 0.5 µM. The same volume of DMSO used in the experimental samples was added to control cells.

### Partial purification protocols

Saturated ammonium sulphate solution was added slowly to 30 ml heat–treated chicken serum at 4°C. Precipitates were redisolved in 3 ml PBS (to give a nominally 10× stock), dialysed overnight against PBS and sterilised by filtration through a 0.45 micron filter [19]. Separate fractions corresponding to proteins precipitated by 0–40%, 40–80% and 80–100% saturated ammonium sulphate were prepared and frozen in aliquots at −20°C until used. DKO-S cells were seeded into the wells of a 24 well plate at 1×10^4^/ml in 2 ml in medium lacking chicken serum and supplemented with 0.1 µg/ml doxycycline to repress clathrin expression. Wells were supplemented with 0.01 ml sterile ammonium sulphate fractions and cell numbers counted after 3 days in those wells showing evidence of cell growth. The ammonium sulphate fraction promoting the strongest growth was loaded onto a Sephadex G-200 column maintained at 4°C and pre-calibrated with high and low molecular weight standards kit (Pharmacia, Sandwich, UK). Elution flow was maintained at 1 ml/min with PBS, collecting 3 ml fractions. Each fraction from the column was filter sterilized and 0.1 ml added to the DKO-S cell bioassay as described above. Positive fractions were pooled, concentrated by ultrafiltration to a final volume of 5 ml and dialysed against 50 mM Tris buffer pH 7.5. The sample was applied to a Mono Q HR 5/5 anion exchange column at room temperature and eluted by Fast-Protein Liquid Chromatography (FPLC) with a linear gradient of 0 −1 M NaCl in 50 mM Tris buffer pH 7.5. Aliquots (0.1 ml) from each fraction were examined by SDS polyacrylamide gel electrophoresis and stained with Coomassie Briliant Blue R-250 (Bio Rad, Hemel Hempstead, UK). The remaining samples of each column fraction were dialysed against PBS, filter sterilised and 0.1 ml aliquots added to the DKO-S bioassay.

### Mass spectrometry of gel excised fractions

Coomassie-stained proteins within the gel pieces were reduced, carboxyamidomethylated, and digested to peptides using trypsin on a MassPrepStation (Waters, Manchester, UK). The resulting peptides were applied to a LC-MS/MS column. For LC-MS/MS, the reverse phase liquid chromatographic separation of peptides was achieved with a PepMap C18 reverse phase, 75 mm i.d., 15-cm column (LC Packings, Amsterdam) on a capillary LC system (Waters) attached to QTof2 (Waters) mass spectrometer or the same column attached to a Dionex Dual Gradient LC system attached to a QSTAR XL (Applied Biosystems, Framingham, MA, USA). The MS/MS fragmentation data achieved was used to search the National Center for Biotechnology Information database using the MASCOT search engine (http://www.matrixscience.com).

### Microarray analysis

We used the 13,209 EST chicken microarray designed by the collaboration between Fred Hutcheson Cancer Research, University of Delaware and ARK Genomics (Roslin Institute) [20]. Full details are available from ArrayExpress (‘ARK-Genomics G. gallus 13 K v 4.0’, accession number: A-MEXP-831). The contents for the array were selected from a clustering of all chicken EST information available in 2003. A single representative of each cluster was selected and supplemented with a range of singleton expressed sequence tags (ESTs) that targeted known genes and some that were known to show differential expression in immune tissues. This array gives a broad coverage of genes from the chicken transcriptome being sourced from over 39 different EST libraries, representing 20 different adult tissues and stages of developmental growth.

Detailed protocols for the microarray analysis are available from the website www.ark-genomics.org (RNA extraction, RNA QC, Amino-allyl labelling, dye coupling, fluorescent labelling QC and hybridisation). Total RNA was prepared from DKO-S and DKO-R cell-lines using two stages, initial extraction was with Trizol (Invitrogen, Paisley, UK) followed by RNAeasy (Qiagen, Crawley, UK). The purified RNAs were quality tested using an Agilent Bioanalyser 2100 (Agilent UK) with all samples having a RIN number in excess of 7.0 which we have found to be acceptable for microarray analysis [20]. The total RNA (20 µg per sample) was tagged with amino-allyl UTP using the Fairplay II kit (Stratagene, UK) according to the manufacturers protocol with the exception that the tagged cDNA was precipitated overnight at −20°C. The precipitate was resuspended in coupling buffer (0.1 M carbonate buffer pH 9.0) and the appropriate Cy3 or Cy5 mono-reactive dye added (GE Healthcare, UK) as required by the experiment. Two fluorescent labels were prepared from each total RNA sample and labelled with different dyes. Samples from DKO-S and DKO-R cells were randomly paired for hybridisation analysis with reciprocal dye combinations for each pair. Hybridisations and washes were carried out overnight on GenTac Hybridisation stations (Genomics Solutions, Huntingdon, UK) as outlined in the protocol. The dried slides were scanned in a Scanarray 5000 XL scanner (GSI Lumonics) at constant laser power of 80% and 78% for Cy3 and Cy5 respectively.

Data was extracted from the slide using BlueFuse software (BlueGnome, Cambridge, UK). Features with poor confidence information (confidence <0.30, flagged D and E) were eliminated from the analysis. M v A plots [where M  =  log_2_ (Cy5/Cy3) and A = 1/2*[log_2_(Cy5) + log_2_(Cy3)] of the data for each slide (data not shown) were suitably linear to require only a simple global normalisation of the data. Analysis was performed using GeneSpring software (Agilent UK) on the fused data (mean of replicate values for features on individual slides) from the BlueFuse program. The data was filtered with a probability of 5% with a false discovery rate (FDR) [21] of 15%. The resulting list of differentially expressed genes was then re-annotated using the EST sequence from the feature to update the information available on each cDNA target on the array.

The microarray data described in this manuscript has been deposited with the Gene Expression Omnibus (GEO) data repository under the accession number GSE57328

### RT-PCR

Total RNA was isolated with the ‘Absolutely RNA’ kit from Stratagene, following the manufacturer's instructions. RNA (1 µg) was used for cDNA synthesis using an Moloney Murine Leukemia Virus (MMLV) reverse transcriptase and random primers (Promega). One µL of RT reaction was used for the PCR following the protocol: 1 min 94°C and 20 cycles of amplification by 1 min 94°C, 1 min 55°C, 1 min 72°C, and finishing with 5 min at 72°C. Amplified products were visualised in 1% agarose with ethidium bromide. The following primers were used:

Actin 5′-attcctatgtgggcgacgag-3′ F, 5′-tggatagcaacgtacatggc-3′ R (259 bps)

SDF-1 5′-gcctgacttaccgatgcc-3′ F, 5′-ggccaactccaaacccatc-3′ R (333 bps)

CXCR4 5′-ccttgccattctggtctgtgg-3′ F, 5′-ccgggcaagacaagtcctacc-3′ R (367 bps)

For real time PCR, the following primers were used:

Cyclophilin A 5′-gcgagaagggatttggctacaaggg-3′F, 5′-ggatttgccaccagtgccgttgtg-3′R (102 bp)

SDF-1 5′-cttcgagagcaacgtggcgag-3′F, 5′-gcacacttgcttgctgttgctc-3′R (112 bp)

CXCR4 5′-ggcagcatggacggtttgg-3′F, 5-gcacggctctccatagtctcc-3′R (108 bp)

CXCR7 5′-cctcgtccagcataaccaatgg-3′F, 5′-ccacgctcatgcatgccag-3′R (113 bp)

Tf 5′-cgagaagggtgatgtggc-3′F, 5′-ggcagagcaactcaaagtc-3′R (118 bp)

TfR 5′-cagttggagtgctggagact-3′F, 5′-gggctggcagaaaccttg-3′R (147 bp)

For amplification with SYBR Green PCR master mix, following the settings recommended by the manufacturer.

### Statistical tests

Apoptotic responses for cells grown either with or without clathrin expression were determined using two-tailed unpaired Student's t-test.

## Results

### Iron-deprivation induces apoptosis in DT40 cells

**Figure 1:**
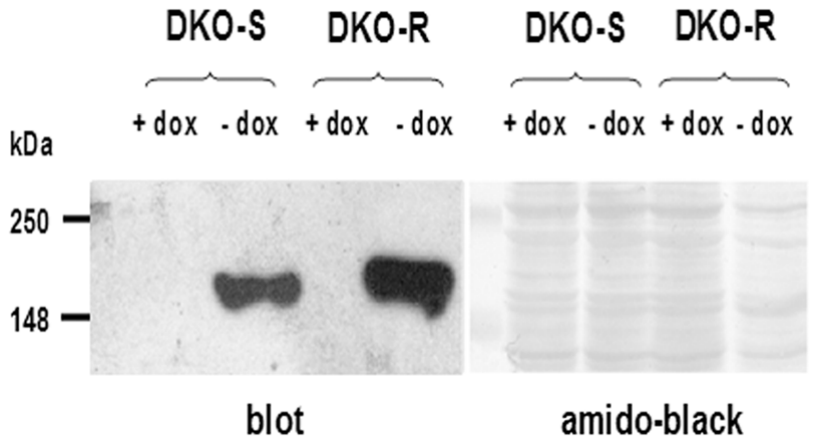
Western blot showing the complete repression of clathrin expression in DKO-S and DKO-R cells when grown in the presence of 0.1 µM doxycycline (dox). Cells were grown for 72 hours in media with or without doxycycline as indicated. Following development of the blot, the nitrocellulose was stained with amido black to detect total protein.

**Figure 2:**
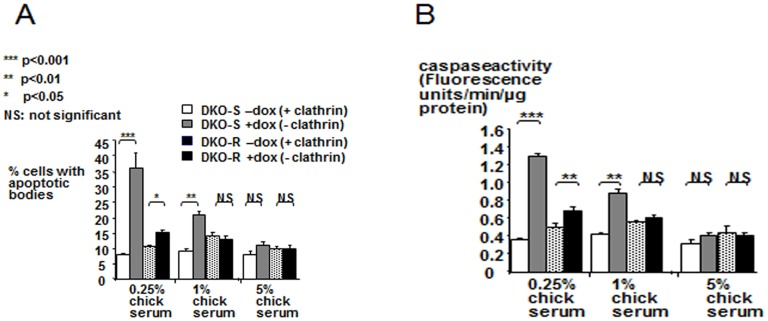
DKO-S cells show a higher apoptotic response to clathrin-depletion than DKO-R cells. (A) The proportion of apoptotic cells and (B) caspase activity were measured as described in materials and methods for DKO-S and DKO-R cells grown with or without 0.1 µM doxycycline (dox) and in media supplemented with increasing concentrations of chicken serum are as indicated. Values are means of three measurements +/− standard deviation. Statistically significant differences, with p values, are indicated.

Addition of 0.1 µM doxycycline to the media of both DKO-S and DKO-R cells completely repressed clathrin expression (Figure 1). DT40 cells are typically grown in media supplemented with 10% foetal-calf serum (FCS) and 1% chicken serum [3]. We previously showed that when DKO-S cells were seeded into this standard medium in the presence of doxycycline, the clathrin-depleted cells died by apoptosis after 3–4 days, reflecting the decline in the level of clathrin over this period. The cells died more quickly when clathrin-depleted DKO-S cells were grown in the presence of 10% FCS, but only 0.25% chicken serum [5]. This suggests that chicken serum contains a factor required by DKO-S cells that becomes growth limiting in the absence of clathrin. To investigate the nature of this factor, we first extended the growth experiments to include a wider range of chicken serum concentrations (0 to 5%) (Figure S1A and S1B). The inhibitory effect of clathrin-depletion on cell growth was particularly dramatic at low levels of chicken serum (0.25%). When seeded into medium lacking chicken serum altogether, DKO-S cells grew noticeably less well even when expressing clathrin, and they needed a seeding density of at least 2×10^4^ cells/ml to grow. In the absence of clathrin, DKO-S cells in this medium died rapidly. By contrast we found that at chicken serum concentrations higher than normally used (up to 5%), DKO-S cells increasingly survived clathrin-depletion (Figure S1A). Presumably, at this high concentration the increased availability of the potential factor helped the cells to survive longer when clathrin expression was repressed. When the same experiment was conducted for the DKO-R cell-line we found a similar pattern, but with the striking difference that DKO-R cells required less chicken serum to survive clathrin-depletion compared to DKO-S cells (Figure S1B). For both clathrin-depleted DKO-S and R cells, reduction in the level of chicken serum increased apoptosis as measured both by caspase activity and nuclear condensation, but the DKO-S cell-line was markedly more sensitive to this effect compared to DKO-R cells as the chicken serum levels were reduced (Figure 2A and B). This suggests that the response of these two cell-lines to clathrin-depletion reflects a similar underlying mechanism, but with a quantitative difference in sensitivity to restriction of the chicken serum factor. Hence, at the level of chicken serum that we routinely used (1%), the DKO-S cells typically died but the DKO-R cells survived in the absence of clathrin.

**Figure 3:**
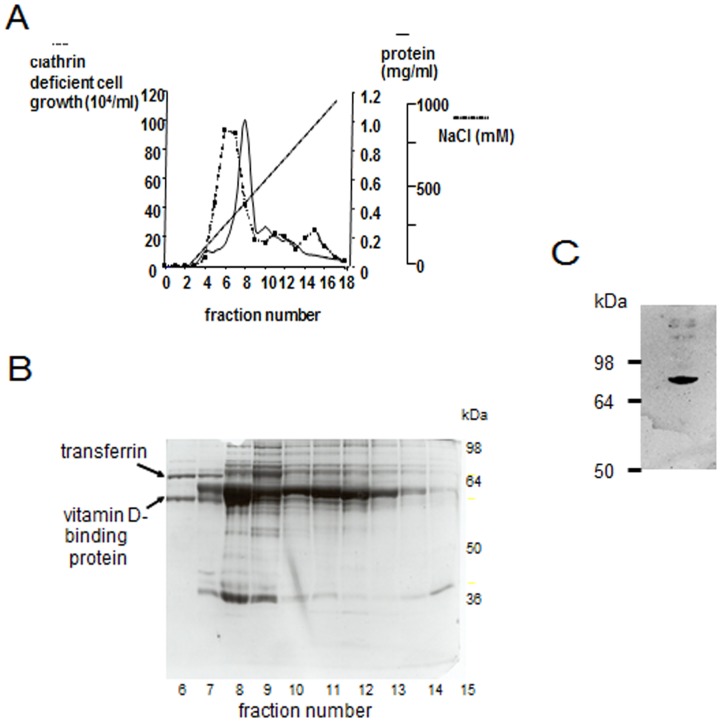
Identification of the putative chicken survival factor as transferrin. (A) FPLC separation of the positive fraction from the gel filtration step showing separation of the main bioactivity peak from major protein fractions. In each case, cell growth after 72 hours was determined as described in materials and methods. (B) SDS polyacrylamide gel electophoresis of the fractions from the FPLC column. Fraction 6, which contains the major bioactivity peak contains only two major proteins identified as vitamin D binding protein and transferrin. (C) Western blot of FPLC fraction 6 using anti-(chicken transferrin) monoclonal antibody.

In order to identify this putative factor, chicken serum was fractionated with ammonium sulphate and the capacity of the different fractions to support growth was examined. For this experiment, DKO-S cells were seeded in a multiwell plate at 1×10^4^ cells/ml in medium without chicken serum (see materials and methods), together with doxycycline to repress clathrin expression. Under these conditions, clathrin-depleted DKO-S cells did not grow (Figure S1A). The ammonium sulphate fractions from chicken serum were added to these cells to give an equivalent chicken serum concentration of about 5%. Only the 40–80% ammonium sulphate fraction supported growth under these conditions (Figure S2A). Gel filtration of this fraction on G-200 Sepharose eluted the growth activity as a broad peak between the 69 and 150 kDa size marker (Figure S2B). Subsequent FPLC ion-exchange chromatography of this gel-filtration fraction produced a single sharp leading peak of growth-supporting biological activity separated from most of the other proteins (Figure 3A). When analysed by SDS PAGE, this peak yielded two major protein bands of apparent molecular weight of about 80 kDa and 60 kDa (Figure 3B). Each of these proteins was separately excised from the gel and analysed by mass spectrometry (MS) sequencing. The lower molecular weight band was identified as vitamin D binding protein [22]. Purified samples of this protein did not support the growth of clathrin-depleted DKO-S cells in the absence of chicken serum (data not shown) even at the highest concentration used. The higher molecular weight band, which did support growth, was identified as chicken transferrin, and this identification was further confirmed by western blotting (Figure 3C).

**Figure 4:**
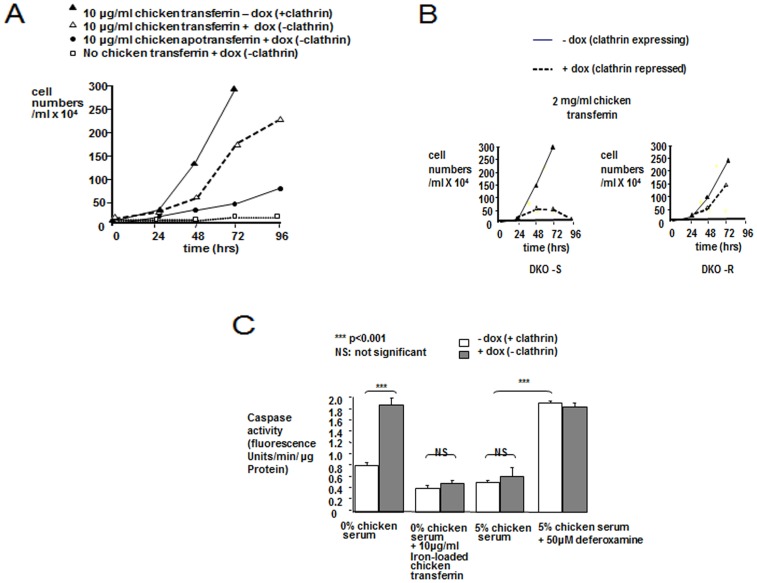
Purified chicken transferrin reproduces the effect of full chicken serum on the cell growth and apoptotic response of DKO-S cells to clathrin-depletion. (A) Fully iron-loaded transferrin, but not apoptransferrin rescues clathrin-depleted DKO-S cells. DKO-S cells were seeded at 2×10^4^ cells/ml in media lacking chicken serum and treated as indicated. Cell growth was monitored as described in Figure 1. (B) Clathrin-depleted DKO-R cells require less chicken transferrin for survival. Cell growth was monitored as described in the legend to Figure 1. (C) Caspase activity in clathrin-expressing or clathrin-depleted DKO-S cells treated with 10 µM iron-loaded transferrin or 50 µM deferoxamine as indicated. Cells were seeded into flasks at (2×10^4^ cells/ml) in treated media and caspase activity measured 72 hours later. Values are means of three measurements +/− standard deviation.

Purified iron-saturated chicken transferrin mimicked the effect of full chicken serum both on cell growth, apoptotic response and sensitivity to clathrin-depletion od DKO-S cells (Figures 4A, B, C). These cells survived clathrin-depletion if they were grown in standard medium without chicken serum, supplemented with concentrations of fully iron-saturated chicken transferrin of 10 µg/ml or higher (Figure 4A). At lower concentrations, (eg 2 µg/ml shown in Figure 4B), clathrin-depleted DKO-S cells died, but DKO-R cells survived (Figure 4B). Using quantitative western blotting, the level of transferrin in our chicken serum was estimated at about 0.5 mg/ml (data not shown) so that 10 µg/ml corresponds to the level of chicken transferrin present in media supplemented with 2% chicken serum. The degree of iron saturation in commercial chicken serum is unknown, but if it is similar to human serum transferrin (about 30% iron-loaded) [23], then this value is consistent with the observed quantitative effects of chicken serum on the two clathrin-depleted cell-lines. Although the DKO-S and -R cells were always grown in media with 10% FCS, it should be noted that, as with other mammalian transferrins [24], bovine transferrin binds relatively weakly to the chicken transferrin receptor (FRW/APJ data not shown). So in these experiments it is likely to be the chicken serum that supplies the majority of the iron. When supplemented with chicken apotransferrin, from which iron had been removed by prior chelation and dialysis [14], growth of clathrin-depleted DKO-S cells was significantly reduced (Figure 4A). It has been reported that FCS contains high levels of iron stores [25], which could supply some intake into the cells *via* coupling to apotransferrin and could explain the residual growth under this condition. By contrast, growth was completely abolished for clathrin-depleted DKO-S cells with apotransferrin (Figure 4A). The role of transferrin and iron in cell survival was confirmed with deferoxamine, a powerful and highly specific iron chelator that is known to prevent iron uptake into cells, and which induced apoptosis of DKO-S cells [26] (Figure 4C).

**Figure 5:**
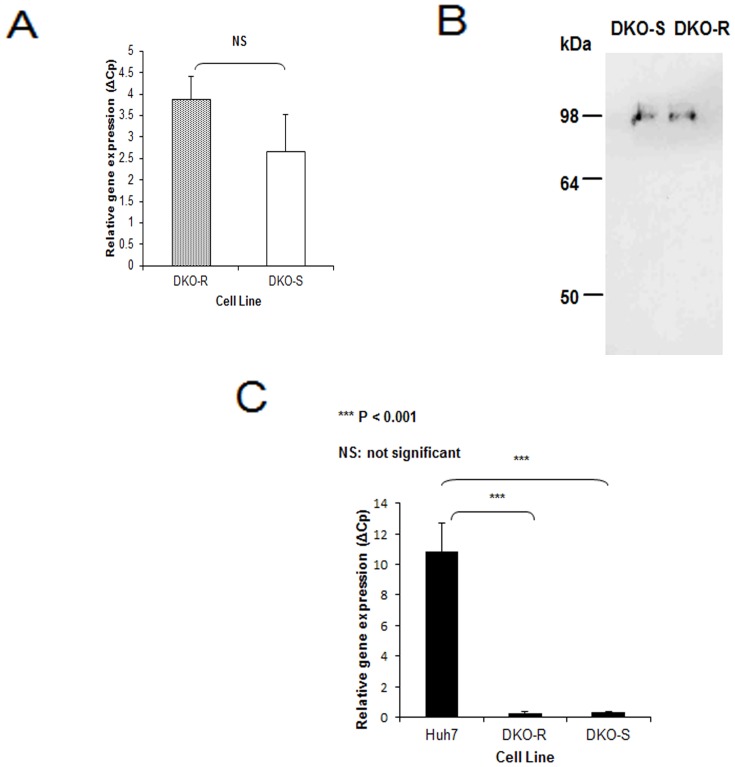
Analysis of the expression of transferrin and its receptor. (A) Quantitative RT-PCT of the transferrin receptor in both cell lines. (B) Western blot for the transferrin receptor in DKO-R and DKO-S cells. (C) Quantitative RT-PCR of transferrin in a control hepatic human cell line (Huh7) and DKO-R and DKO-S cells. Statistically significant differences, with p values, are indicated.

Does the differential survival of clathrin-depleted DKO-S and R cells reflect differences in transferrin receptor (TfR) expression? A quantitative RT-PCR analysis showed similar levels of TfR mRNA in DKO-R and DKO-S (Figure 5A). Likewise, western blotting confirmed similar levels of TfR protein in the two cell-lines (Figure 5B). These results are consistent with our previous report showing that the rates of transferrin internalisation into DKO-S and DKO-R cells are similar and reduced to similar levels when clathrin is depleted [8]. An alternative possibility is that DKO-R cells synthesise their own transferrin, which could then support survival. However, neither cell line expresses detectable levels of transferrin mRNA (Figure 5C) so the difference between DKO-S and DKO-R does not rely on changes in expression of the transferrin iron uptake pathway. Hence, the lower apoptotic sensitivity shown by the DKO-R cells must result from an additional mechanism.

### Endogenous expression of SDF-1 is responsible for DKO-R resistance to clathrin-depletion-induced apoptosis

**Figure 6:**
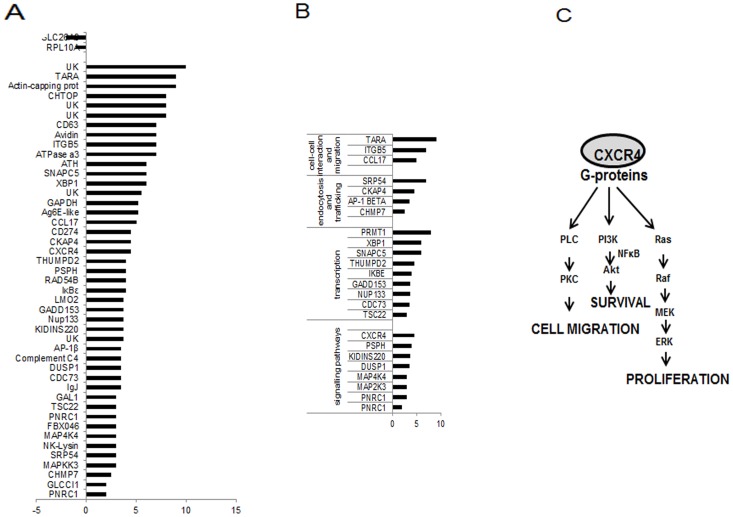
Microarray analysis of the gene expression profiles of DKO-s and DKO-R cells. (A) differentially expressed genes ordered in descending significance according to the P value UK: unknown. (B) functional clusters of genes of processes implicated in cell fate. (C) depiction of the CXCR4 signalling pathway.

**Figure 7:**
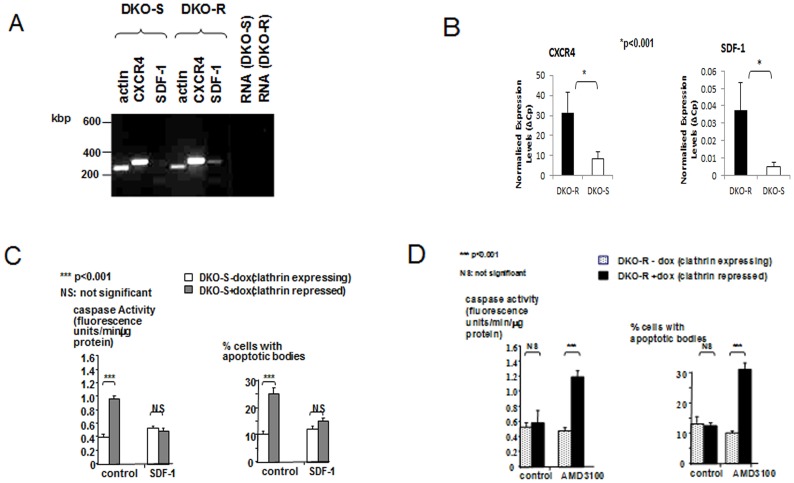
RT-PCR for CXCR4, SDF-1 and CXCR7 in DKO-S and DKO-R cells. (A) standard RT-PCR as described in materials and methods followed by agarose gel migration. Controls (RNA) for genomic DNA contamination in which the RT-PCR reaction was conducted without prior treatment with reverse transcriptase are shown. (B) real time PCR from cDNA obtained from both cell-lines, results are expressed as ΔCp normalising the levels of expression to Cyclophilin A. (C) Caspase activity and percentage of apoptosis of clathrin-expressing and clathrin-repressed DKO-S cells in the presence or absence of 20 nM recombinant human SDF-1α. Values are means of four determinations +/− standard deviation. (D) Caspase activity and percentage of apoptosis of clathrin-expressing and clathrin-repressed DKO-R cells in the presence or absence of 5 µM AMD3100. In both (C) and (D), cells were grown in standard DT40 medium with 1% chicken serum. Values are means of four determinations +/− standard deviation. Statistically significant differences, with p values, are indicated.

Since the distinctive phenotypes of the DKO-S and R cells are stable [5], they could reflect changed gene expression profiles. To search for the pathway responsible for cell survival, we compared the global patterns of mRNA expression between the two cell-lines using an EST chicken microarray [20] (see Materials and Methods). There was a limited number of statistically significant differences between DKO-S and DKO-R cells (Table S1). This is to be expected, since the DKO-R cell-line derives from the DKO-S parent [5]. One ribosomal protein was up-regulated in DKO-S cells, but the physiological significance of this is unclear. On the other hand, several genes were up-regulated in DKO-R cells by a factor of two-fold or more compared to DKO-S cells (Figure 6 and Table S1). Figure 6A shows these genes in decreasing order of significance and from these, groups of functionally related genes have been highlighted in Figure 6B. Strikingly, 65% of the up-regulated genes in DKO-R cells whose function is known, are components of the pathways implicated in signalling, trafficking and cell interaction. Of these genes, the chemokine receptor CXCR4 was of particular interest. CXCR4 is a serpentine G-protein coupled receptor that is commonly expressed on lymphocyte cell-lines, including B and pre-B-cells [27]. When activated by its ligand, stromal cell-derived factor 1 (SDF-1, CXCL12), the CXCR4 receptor stimulates calcium entry, MAP kinase and Akt-dependent pathways [28] (Figure 6C). Indeed, in previous work we found constitutively active Akt and ERK1/2 in DKO-R cells [5], which is consistent with the activation of CXCR4 signalling. These pathways in turn modulate actin cytoskeletal rearrangements, enhanced integrin expression, and stimulate chemotaxis towards SDF-1 secreting cells [29], [30], [31], Several genes potentially connected to these pathways were up-regulated in DKO-R cells including MAP kinase kinase 3, integrin β5, actin capping protein and Tara, a regulator of actin rearrangement (Figure 6 and Table S1). Crucially in the present context, SDF-1/CXCR4 signalling has been implicated in cell survival with a predominantly anti-apoptotic effect in lymphocyte cell-lines [32], [33], [34]. SDF-1 was not present in the microarray data set, so to confirm and extend the microarray results, we examined CXCR4 and SDF-1 mRNA expression in DKO-S and DKO-R cells by qualitative and quantitative RT-PCR. Both cell-lines expressed CXCR4, although there was a higher expression in DKO-R compared to DKO-S cells and SDF-1 was detectable in DKO-R cells, but not DKO-S (Figure 7A). Consistent with the microarray data and the qualitative RT-PCR, there was a significant increase in CXCR4 mRNA in DKO-R cells compared to DKO-S cells. Strikingly, SDF-1 expression showed an 7.5 fold increase in DKO-R cells compared to DKO-S (Figure 7B). Wild-type DT40 cells expressed SDF-1 mRNA at a level comparable to the DKO-S cell-line and the differential expression of CXCR4 and SDF-1 in these cell lines was independent of the presence of clathrin in the cells (Figure S3).

It has previously been shown that recombinant human SDF-1 can activate CXCR4 signalling on DT40 cells [35]. Treatment of clathrin-depleted DKO-S cells with recombinant human SDF-1α significantly reduced apoptosis at a chicken serum concentration (1%) that was normally lethal for these cells (Figure 7C). The selective activation of CXCR4 signalling in DKO-S cells led to a more DKO-R-like phenotype in their response to clathrin-depletion. The reciprocal experiment was performed with the drug AMD3100 which is a highly selective inhibitor of CXCR4 signalling [36], [37]. At a concentration of 5 µg/ml, AMD3100 did not affect apoptotic behaviour of clathrin-expressing DKO-R cells in medium containing 1% chicken serum. However, when clathrin expression was repressed in DKO-R cells, under conditions where they normally survived (ie 1% chicken serum), the AMD3100-treated, clathrin-depleted DKO-R cells now underwent apoptosis (Figure 7D).

**Figure 8:**
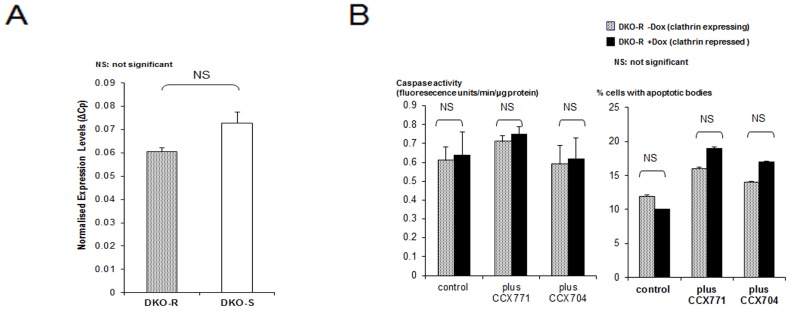
CXCR7 signaling is not responsible for the apoptotic-resistace shown by clathrin-depleted DKO-R cells. (A) Quantitative RT-PCR for CXCR7 in both cell lines. (B) Caspase activity and percentage of apoptosis of clathrin-expressing and clathrin-repressed DKO-R cells in the presence or absence of 0.5 µM CCX771 and inactive analog CCX704. Cells were grown in standard DT40 medium with 1% chicken serum. Values are means of four determinations +/− standard deviation.

Although SDF-1 is the only ligand for CXCR4, it can also bind another receptor in this family, CXCR7 [38]. In order to assess the participation of CXCR7-mediated signalling, we examined CXCR7 expression in both cell lines. The levels of CXCR7 were similar between DKO-R and DKO-S and approximately 100 fold lower than the levels of CXCR4 (note the different ordinance scales in Figure 8A compared to Figure 7B). In order to assess the potential role of CXCR7 we investigated the effect of a highly specific inhibitor: CCX771 [39] and an inactive close analogue compound CCX704 on the apoptotic response of DKO-R cells to clathrin-depletion. In contrast to the CXCR4 inhibitor AMD3100, neither CCX771 nor CCX704 had any effect on caspase activity (Figure 8B). Although a small increase in apoptosis was observed, this was the same with both the active and inactive CXCR7 antagonists and was not accompanied by an increase in caspase activity. Taken together, our data indicates that DKO-R cells, unlike the DKO-S cells, can operate an anti-apoptotic autocrine pathway, due to the simultaneous expression of SDF-1 ligand and its receptor CXCR4.

## Discussion

Our initial observation that clathrin-depletion in DKO-S cells induced apoptosis is surprising because in mammalian cells, RNAi-mediated clathrin knockdown does not produce the same effect [10], [40]. However, proliferation and survival of some tumour cell lines are correlated with transferrin uptake [41]. In particular, iron restriction is known to induce apoptosis in activated T-lymphocytes and the promyelocytic cell-line HL60 [42]. Furthermore, a monoclonal antibody that blocks transferrin binding to its receptor induced apoptosis in adult T-cell leukaemia cells [43]. As with DT40, these are all fast growing lymphocyte-derived cells, suggesting that a relatively high apoptotic sensitivity to iron deficiency may be a common feature of this type of cell-line. A comparison of several human and mouse cell-lines with regards to sensitivity to iron deprivation showed that cell-lines of haematopoietic origin are indeed particularly sensitive to iron restriction. Interestingly HeLa cells, commonly used in assays of clathrin-function have a relatively high resistance to apoptotic stimuli [44].

Iron is a cofactor for key enzymes required for purine and pyrimidine synthesis, the tricarboxylic acid cycle and the mitochondrial electron transport chain [45]. Thus in fast growing cells, iron deficiency may limit both DNA synthesis and the capacity to generate ATP from aerobic respiration. Iron restriction reduces transcription of the cell cycle control protein p21(WAF1/CIP1) [46], downregulates anti-apoptotic Bcl-2, up-regulates the pro-apoptotic Bax [47] and activates a mitochondrial caspase pathway [48]. Clathrin-depletion in the DT40 cells reduced, but did not abolish transferrin uptake [5], [8]. This clathrin-independent residual transferrin uptake probably accounts for the survival of clathrin-depleted DKO-S cells when grown in media with high levels of chicken serum. We have found that some batches of chicken serum are better than others at supporting the growth of clathrin-depleted DKO-S cells [8], and this correlates with the level of transferrin (APJ, data not shown). Additionally, in cells with increased requirements for iron due to their rapid proliferation rate, such as neoplastic cells, alternative routes of iron uptake to transferrin have been observed. Thus non-receptor-mediated pinocytosis has been suggested as a significant mechanism of iron uptake in human hepatoma and melanoma cell-lines [49].

The different apoptotic thresholds shown by clathrin-depleted DKO-S and DKO-R cells is a notable example of how cells can modulate their apoptotic response to iron deficiency depending on the activity of other signals. We propose that the DKO-R cell-line has induced an autocrine loop mediated by the anti-apoptotic chemokine SDF-1 and its receptor CXCR4. Thus the DKO-R cells have enhanced an important survival pathway that helps to damp down the apoptotic signal generated by partial iron restriction. Our work suggests that SDF-1/CXCR4 autocrine signalling can play an important role in regulating tumour survival [32] and is consistent with the observation that activation of this pathway reduces apoptosis in serum-starved murine embryonic stem cells [12]. High expression of CXCR4 was associated with carcinoma, migration, proliferation and metastasis [50]. The DT40 cell-line was derived from a pre B-cell [2] and indeed, SDF-1 was initially named ‘Pre B-cell growth-stimulating factor’ in recognition of its role in promoting the survival of pre B-cells *in vitro* [51]. In T cells, activated CXCR4 stimulates both MAP kinase and Akt-dependent pathways [34], [52]. We showed that phospho-Akt is much more abundant in DKO-R than DKO-S and though a decrease is observed in both lines after clathrin depletion, this decrease is less prominent in DKO-R and Akt activity remains much higher than in DKO-S. We also examined ERK1/2 showing that it is constitutively active in DKO-R cells [5]. As Akt and ERK are pivotal to the CXCR4 signalling pathway, and since CXCR4 only binds SDF-1 [53], the constitutive stimulation of these enzymes in DKO-R cells is consistent with an autocrine effect of endogenously synthesised SDF-1. In agreement with our observations, a dramatic increase in Akt and ERK1/2 phosphorylation in response to SDF-1 stimulation has been noted in MEF cells and this activation was inhibited by the CXCR4 inhibitor AMD3100 but not by a CXCR7 inhibitor [54].

SDF-1 activated CXCR4 receptors modulate integrin signalling, promote chemotaxis towards SDF-1 secreting cells and induce major actin cytoskeletal rearrangements [28]. It is possible that some of these changes could affect additional aspects of the DKO-R phenotype. For example, it has been observed that receptor-mediated endocytosis of the B-cell receptor (but not other receptors) is significantly less clathrin-dependent in DKO-R cells compared to DKO-S cells [8]. It will now be interesting and important to investigate whether SDF-1 signalling can explain this unusual result. Evidently, the altered expression of only a small number of genes is enough to produce the DKO-R phenotype and several of these are directly related to the SDF-1 signal pathway. In prostate tumour cell-lines, SDF-1 signalling up-regulates the expression of CXCR4 and other downstream targets of its signalling pathway [55]. If this is also true for DT40, then the primary change that produced the DKO-R phenotype could have been an up-regulation of SDF-1 expression. This is supported by our observation that wild-type DT40 cells do not express SDF-1 (Figure S3). The DKO-R cells were originally isolated from an overgrown population of DKO-S cells, and it seems likely that this condition selected for an enhanced anti-apoptotic phenotype. We have subsequently identified other cells derived from DKO-S that are similarly resistant to clathrin-depletion, although in these cases, the molecular mechanism has not been investigated. It appears that such variants are not uncommon in DT40 populations and this may in turn have important implications for the use of DT40 to study apoptotic pathways.

Our results provide a worked example of how the phenotypic consequences of gene inactivation are contingent upon the wider physiological background and how this needs to be taken into account when interpreting gene knockout experiments. Furthermore, many tissue-culture cells possess great plasticity to adapt to their environment *via* genetic changes, and these adaptations can have an impact on the phenotype generated by research manipulations. Our results further emphasise that cell survival depends on a balance of positive and negative signals that can be independently modulated. A number of therapeutic strategies for the treatment of leukaemia and other lymphocyte-derived tumours have been proposed and are currently undergoing clinical trials. Several of these aim to stimulate apoptosis by blocking transferrin endocytosis [43], [56], or prevent iron uptake using chelating agents [57]. Moreover, the up-regulation of SDF-1/CXCR4 expression is a known mechanism used by some tumour cells that contributes to chemotherapy resistance [30]. In this context, the relative ease with which the DKO-R cells emerged from the DKO-S background should raise a cautionary note, as it implies a simple mechanism by which cells can escape from such therapies. Adaptive changes based on CXCR4-SDF-1 regulation and signalling may be important factors in the resistance to chemotherapy.

## Supporting Information

Figure S1 **Figure S1A: growth of DKO-S cells in increasing levels of chicken serum, as indicated.** **Figure S1B**: growth of DKO-R cells in increasing levels of chicken serum as indicated.
(TIF)

Figure S2 **Cell growth of clathrin-deficient DKO-S cells.** (A) in ammonium sulfate fractionated chicken serum. (B) in G-200 Sepharose filtered fractions of the 40–80% ammonium sulphate fraction.
(TIF)

Figure S3 **Effect of doxycycline on expression of CXCR4 and SDF-1.** Wild-type DT40 cells as well as the DKO-R and DKO-S clones were grown with or without 0.1 µM doxycycline (DOX) for 72 hours prior RNA extraction. The levels of CXCR4 and SDF-1 expression were assessed by qRT-PCR normalising to cyclophilin A.
(TIF)

Table S1 **Microarray data showing genes differentially expressed in DKO-S and DKO-R cells.** For genes upregulated in DKO-R cells, CXCR4 and other components of pathways known to be modified by activated CXCR4 signalling are shaded.
(XLS)
