# Supplementary figures and images for: SDF-1 Chemokine Signalling Modulates the Apoptotic Responses to Iron Deprivation of Clathrin-Depleted DT40 Cells
Source: PLoS One. 2014 Aug 27;9(8):e106278. doi: 10.1371/journal.pone.0106278 (PMC4146602; doi:10.1371/journal.pone.0106278)

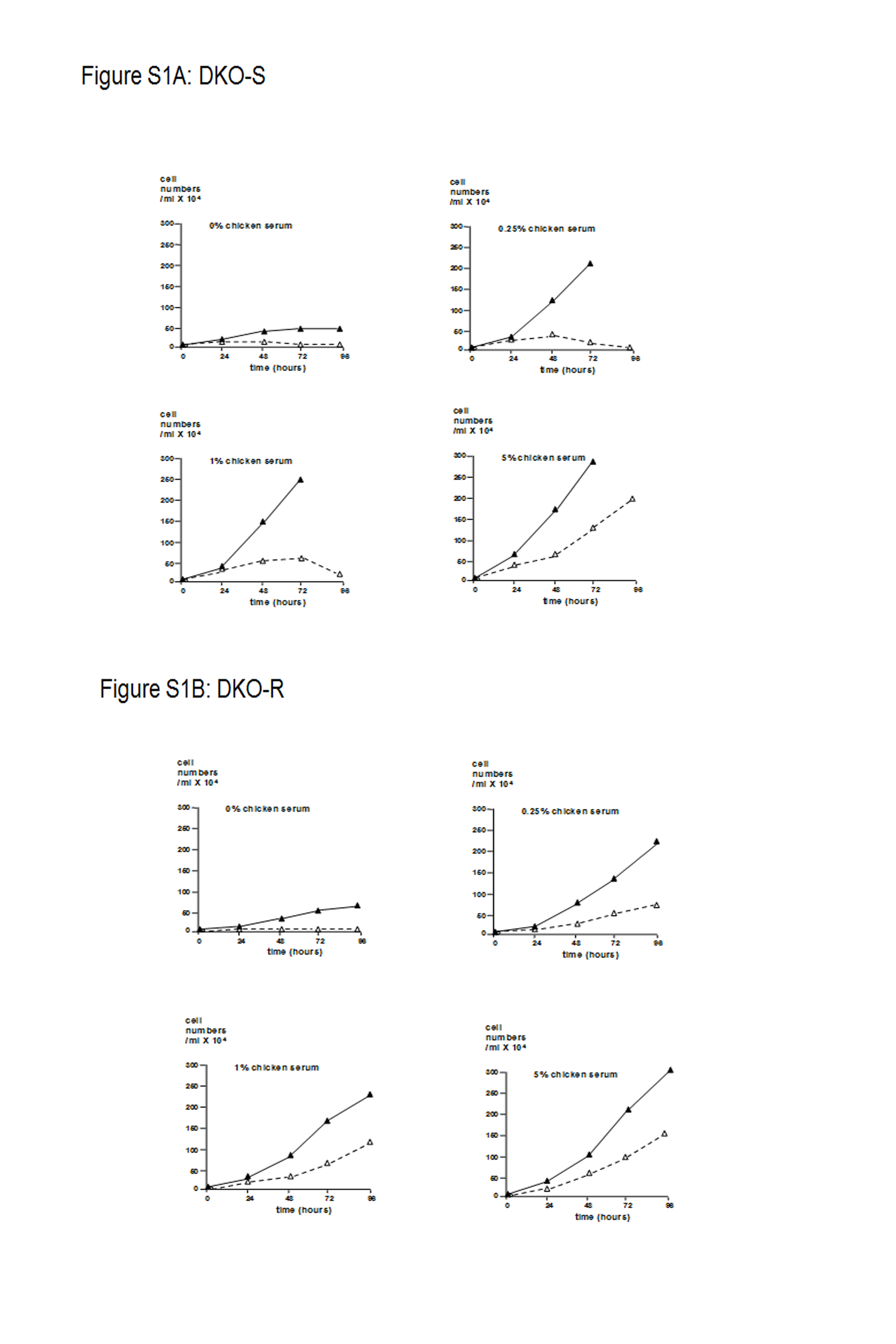

Supplement: Figure S1 — A: growth of DKO-S cells in increasing levels of chicken serum, as indicated. Figure S1B: growth of DKO-R cells in increasing levels of chicken serum as indicated. (TIF) [file pone.0106278.s001.tif]

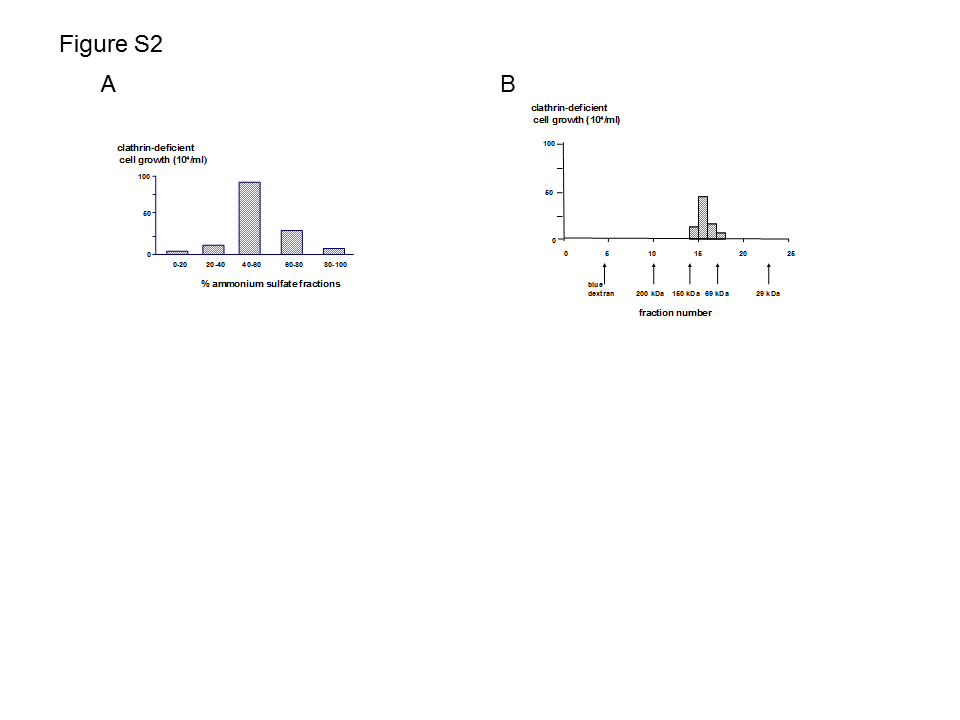

Supplement: Figure S2 — Cell growth of clathrin-deficient DKO-S cells. (A) in ammonium sulfate fractionated chicken serum. (B) in G-200 Sepharose filtered fractions of the 40–80% ammonium sulphate fraction. (TIF) [file pone.0106278.s002.tif]

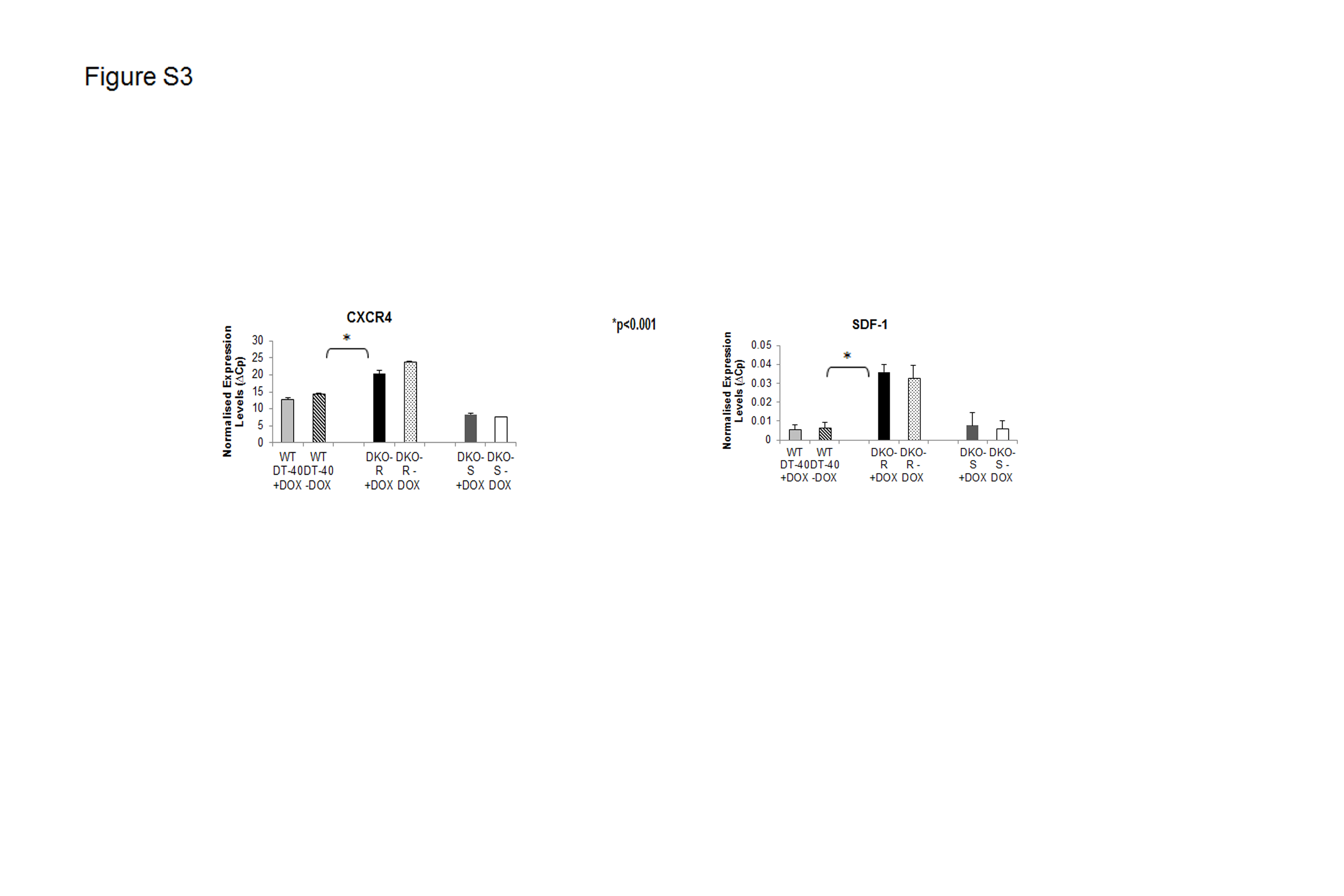

Supplement: Figure S3 — Effect of doxycycline on expression of CXCR4 and SDF-1. Wild-type DT40 cells as well as the DKO-R and DKO-S clones were grown with or without 0.1 µM doxycycline (DOX) for 72 hours prior RNA extraction. The levels of CXCR4 and SDF-1 expression were assessed by qRT-PCR normalising to cyclophilin A. (TIF) [file pone.0106278.s003.tif]
